# Effectiveness and Impact of Autumn 2023 COVID‐19 Vaccination in Preventing Hospitalizations in Navarre, Spain, October 2023 to September 2024

**DOI:** 10.1111/irv.70163

**Published:** 2025-09-13

**Authors:** Iván Martínez‐Baz, Camino Trobajo‐Sanmartín, Miguel Fernández‐Huerta, Ana Navascués, Aitziber Echeverria, Nerea Egüés, Noelia Vera‐Punzano, María Eugenia Portillo, Guillermo Ezpeleta, Jesús Castilla

**Affiliations:** ^1^ Instituto de Salud Pública de Navarra Pamplona Spain; ^2^ CIBER Epidemiología y Salud Pública (CIBERESP) Madrid Spain; ^3^ Instituto de Investigación Sanitaria de Navarra (IdiSNA) Pamplona Spain; ^4^ Clinical Microbiology Department Hospital Universitario de Navarra Pamplona Spain; ^5^ Clinical Microbiology Department Hospital Universitario de Gran Canaria Doctor Negrín Las Palmas de Gran Canaria Spain

**Keywords:** case–control study, COVID‐19, COVID‐19 vaccine, hospitalization, impact, vaccine effectiveness

## Abstract

**Objective:**

We aimed to estimate autumn 2023 COVID‐19 vaccine effectiveness (CVE) in preventing hospitalizations due to COVID‐19 until September 2024.

**Methods:**

We performed a test‐negative case–control study nested in the cohort of adults aged ≥ 45 years with indication of autumn 2023 COVID‐19 vaccination in Navarre, Spain. The study included patients hospitalized for severe acute respiratory infection (SARI) and tested by polymerase chain reaction between October 2023 and September 2024. The COVID‐19 vaccination statuses in the current and previous seasons were compared between confirmed COVID‐19 cases and test‐negative controls. CVE was estimated as (1 − adjusted odds ratio) × 100.

**Results:**

Of 4051 SARI hospitalized patients included in the study, 474 (12%) were confirmed for COVID‐19. CVE to prevent COVID‐19 hospitalizations was 32% (95% confidence interval [CI], 11%–48%) on average for the year and 38% (95% CI, 17%–54%) among people aged ≥ 65 years. However, estimates for current‐season vaccination were 51% (95% CI, 30%–66%), 50% (95% CI, 16%–70%), and 0% (95% CI, −42% to 30%) for 7 to 89, 90 to 179, and ≥ 180 days between vaccination and COVID‐19 diagnosis, respectively. The residual effect of previous‐season vaccination was not statistically significant (14%; 95% CI, −20% to 39%). CVE was moderate in preventing COVID‐19 hospitalizations between October 2023 and March 2024 (50%; 95% CI, 28%–65%), and null between April and September 2024 (6%; 95% CI, −41% to 38%). The vaccine averted 19% of COVID‐19 hospitalizations. On average, 963 doses of vaccine were necessary to prevent one COVID‐19 hospitalization.

**Conclusions:**

CVE was moderate in preventing COVID‐19 hospitalizations during the 2023–2024 season, but decreased 6 months after vaccination.

## 1 Introduction

The SARS‐CoV‐2 continued to circulate around the world during the 2023–2024 season and produced two epidemic waves in many European countries, one in winter and one in summer of 2024 [1, 2]. The incidence of COVID‐19 hospitalization remained higher among individuals aged ≥ 65 years. In European countries, the SARS‐CoV‐2 Omicron XBB.1.5 variant predominated during the early 2023–2024 season, and it was gradually replaced by the BA.2.86 and KP.3 variants [1, 2, 3].

The European Medicines Agency authorized three adapted XBB.1.5 COVID‐19 vaccines (Comirnaty Omicron XBB.1.5, Spikevax XBB.1.5, and Nuvaxovid XBB.1.5) [4], but in the autumn 2023 vaccination campaign in Spain, only Comirnaty Omicron XBB.1.5 was used [5].

In the Northern Hemisphere, early COVID‐19 vaccine effectiveness (CVE) estimates during 2023–2024 showed moderate effect in preventing COVID‐19 hospitalizations [6, 7, 8, 9, 10]. However, since the circulation of the SARS‐CoV‐2 continued during spring and summer and the variants involved changed, it is of interest to evaluate the CVE during the entire season. Studies in previous seasons showed notable effects of COVID‐19 vaccination in preventing the infection and severe disease and the decline of these effects several months after the vaccine booster [11, 12, 13, 14, 15, 16, 17]. The impact of the COVID‐19 vaccination campaign in a population depends not only on the CVE, but also on the COVID‐19 epidemiology, vaccination coverage, and vaccination strategy [18].

We aimed to estimate the CVE and impact of the booster dose received in the autumn of 2023 in preventing hospitalizations due to confirmed COVID‐19 during the 12‐month period from October 2023 to September 2024, as well as the possible remaining effect of the booster dose received in the 2022–2023 season.

## 2 Methods

### 2.1 Study Population and Design

This study was performed in the Navarre region, Spain, where the Health Service provides free healthcare attention at the point of use to residents (680,000 inhabitants, approximately). From October to November 2023, a booster dose of the XBB.1.5 monovalent COVID‐19 vaccine (Comirnaty Omicron, Pfizer) was offered free of charge to the target population that included people aged ≥ 60 years and people from 6 months to 59 years old with major risk conditions [19]. In the autumn of 2022, a booster with the BA.4/5 COVID‐19 vaccine (Comirnaty Omicron, Pfizer) had been offered to the same target population.

The study population included the adults who were covered by the Navarre Health Service. A test‐negative case–control study nested in this population cohort was conducted in severe acute respiratory infection (SARI) patients hospitalized for 24 h or more [20], who were tested for COVID‐19 between October 2023 and September 2024.

### 2.2 Sources of Information and Variables

The present study was based on the enhanced surveillance of SARI patients hospitalized in Navarre. The protocol for clinical management in hospitals established early detection and swabbing of all patients with SARI in emergency rooms before hospital admission.

All samples were tested by real‐time reverse transcription–polymerase chain reaction (RT‐PCR). A random sample of SARS‐CoV‐2–positive specimens with a cycle threshold ≤ 30 was selected for viral whole genome sequencing using the Illumina COVIDSeq Assay (Illumina Inc., San Diego, USA) and the Illumina MiSeq instrument. Subsequently, results were analyzed using the pipeline DRAGEN COVID, included in the Illumina BaseSpace. Sequencing results were also deposited in the Global Initiative on Sharing All Influenza Data (GISAID) database.

Cases were SARI patients hospitalized due to COVID‐19 confirmed by RT‐PCR, and controls were SARI hospitalized patients who tested negative for COVID‐19. Patients who were not targeted for COVID‐19 vaccination, aged < 45 years, hospitalized for less than 24 h, or hospitalized before respiratory symptoms onset were excluded. In the main analysis, patients confirmed for influenza virus were excluded from the control group, since the association between COVID‐19 and influenza vaccinations could bias CVE estimates [21]. Patients confirmed for respiratory syncytial virus were not excluded from the control group because the vaccines against this virus were not yet used in the study season.

The age, sex, presence of major chronic conditions, nursing home residence, and month of sample collection of the study population were obtained from the electronic records of primary healthcare. The major chronic conditions considered were heart disease, respiratory disease, renal disease, cancer, diabetes mellitus, liver cirrhosis, dementia, stroke, immunocompromised, rheumatic disease, and body mass index ≥ 40 kg/m^2^, and these conditions were codified according to the *International Classification of Primary Care*, second edition [22].

The COVID‐19 vaccination statuses in the current (2023–2024) and previous (2022–2023) seasons were obtained from the online regional vaccination registry. Individuals were considered to be protected 7 days after vaccine administration. All patient information was linked using an individual identification number.

### 2.3 Statistical Analysis

Characteristics of cases and controls were compared by *χ* ^2^ test. Logistic regression was used to calculate the odds ratios (ORs) with their 95% confidence intervals (CIs). Models were adjusted for sex, age group (45–64, 65–84, and ≥ 85 years), presence of major chronic conditions, nursing home residence, and month of sample collection. The CVE was estimated as a percentage: (1 − OR) × 100.

Three models were considered. Model 1 considered only the current season COVID‐19 vaccine. Model 2 evaluated combinations of the current and previous seasons vaccination in three categories: unvaccinated in the current and previous seasons as the reference category, vaccinated in the previous season and unvaccinated in the current one, and current season vaccinated regardless of the previous dose. Model 3 was based on Model 2 and distributed people vaccinated in the current season into three categories depending on the time since the booster dose administration (7–89, 90–179, and ≥ 180 days).

Stratified analyses were done for adults aged ≥ 65 years and by periods: October 2023 to March 2024, which included the winter epidemic wave, and April to September 2024, which included the summer wave.

A sensitivity analysis including influenza confirmed patients in the control group was performed to evaluate the possible bias due to this inclusion [21].

The number of hospitalizations prevented by COVID‐19 vaccination in the study population was calculated by multiplying the observed number of vaccinated cases by (1 − OR)/OR. The population cohort covered by the Navarre Health Service was used to calculate incidence rates per 100,000 inhabitants. The number of people vaccinated necessary to prevent one hospitalization was calculated by dividing the actual number of people vaccinated with the COVID‐19 booster dose by the estimated number of hospitalizations averted.

## 3 Results

### 3.1 Characteristics of Study Patients

The study cohort included 219,136 people aged ≥ 45 years with indication for COVID‐19 vaccination, and 49.0% of them had received the boosted dose in the 2023–2024 season. The cumulative incidence of hospitalizations due to COVID‐19 was 216.3 per 100,000 people in the entire season and 102.2 and 114.1 per 100,000 people from October 2023 to March 2024 and from April to September 2024, respectively.

**FIGURE 1:**
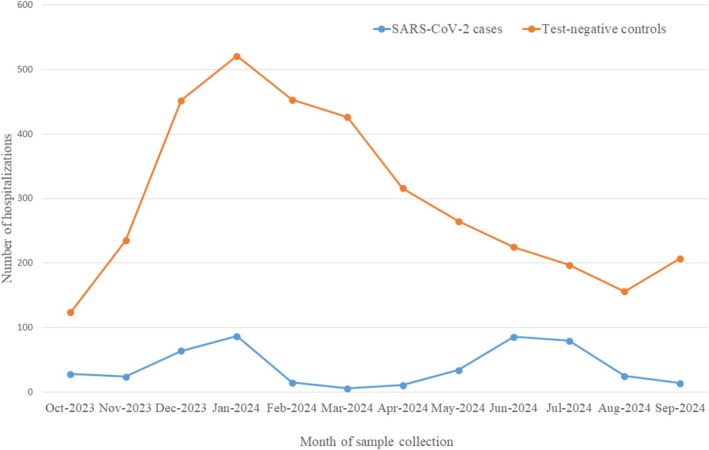
SARS‐CoV‐2 confirmed cases and test‐negative controls recruited by month of sample collection in Navarre, Spain, October 2023 to September 2024.

A total of 4051 SARI patients hospitalized from October 2023 to September 2024 were tested for COVID‐19 by RT‐PCR and included in the test‐negative case–control study; 474 (12%) of them were confirmed for COVID‐19, and 3577 tested negative for COVID‐19 and influenza. COVID‐19 cases were distributed in two waves, with peaks in January and June–July 2024 (Figure 1).

COVID‐19 cases were more likely aged ≥ 85 years than test‐negative controls (41% vs. 35%, *p* = 0.020). No statistically significant differences were observed by sex, nursing home residence, and presence of major chronic conditions (Table 1).

**TABLE 1:** Characteristics of SARS‐CoV‐2 hospitalized cases and test‐negative controls included in the study in Navarre, Spain, October 2023 to September 2024.

	SARS‐CoV‐2 hospitalized cases	Test‐negative controls	*p*
	n (%)	n (%)	
**Age groups, years**			0.020
45–64	48 (10)	483 (14)	
65–84	233 (49)	1840 (51)	
≥ 85	193 (41)	1254 (35)	
**Sex**			0.065
Male	276 (58)	1922 (54)	
Female	198 (42)	1655 (46)	
**Major chronic condition**			0.208
No	24 (5)	235 (7)	
Yes	450 (95)	3342 (93)	
**Nursing home residence**			0.054
No	424 (89)	3085 (86)	
Yes	50 (11)	492 (14)	
**Current season vaccination**			0.012
No	194 (41)	1253 (35)	
Yes	280 (59)	2324 (65)	
**Time since current season vaccination**			<0.001
Unvaccinated	194 (41)	1253 (35)	
Vaccinated 7–89 days before	82 (17)	784 (22)	
Vaccinated 90–179 days before	27 (6)	812 (23)	
Vaccinated ≥ 180 days before	171 (36)	728 (20)	
**Current and previous vaccination**			0.036
Unvaccinated in current and previous season	108 (23)	723 (20)	
Previous dose and no current	86 (18)	530 (15)	
Current regardless previous dose	280 (59)	2324 (65)	
**Month of sample collection**			<0.001
October 2023	28 (6)	124 (4)	
November 2023	24 (5)	235 (7)	
December 2023	64 (14)	452 (13)	
January 2024	87 (18)	521 (15)	
February 2024	15 (3)	453 (13)	
March 2024	6 (1)	426 (12)	
April 2024	11 (2)	316 (9)	
May 2024	34 (7)	265 (7)	
June 2024	86 (18)	225 (6)	
July 2024	80 (17)	197 (6)	
August 2024	25 (5)	156 (4)	
September 2024	14 (3)	207 (6)	
**Total**	474 (100)	3577 (100)	

A lower proportion of cases than controls had received the COVID‐19 vaccine in the current season (59% vs. 65%, *p* = 0.012); however, the proportion of those who had received a vaccine dose in the previous season only was similar (18% vs. 15%) (Table 1). In COVID‐19 vaccinated cases and controls, the median time since vaccination was 215 days (interquartile range [IQR], 78.25–252.75) and 128 (IQR, 71–205), respectively.

Whole genome sequencing of SARS‐CoV‐2 from 223 cases diagnosed between January and July 2024 revealed that 50% (111/223) belonged to the JN.1 variant and 35% (79/223) to the KP.3 variant, with 52 of them (66%) KP.3.1.1. The JN.1 variant was dominant until April 2024 and was gradually replaced by the KP.3 variant. Other Omicron subvariants, such as LB.1, HV.1, and EG.5.1, were occasionally detected.

### 3.2 COVID‐19 Vaccine Effectiveness

**FIGURE 2:**
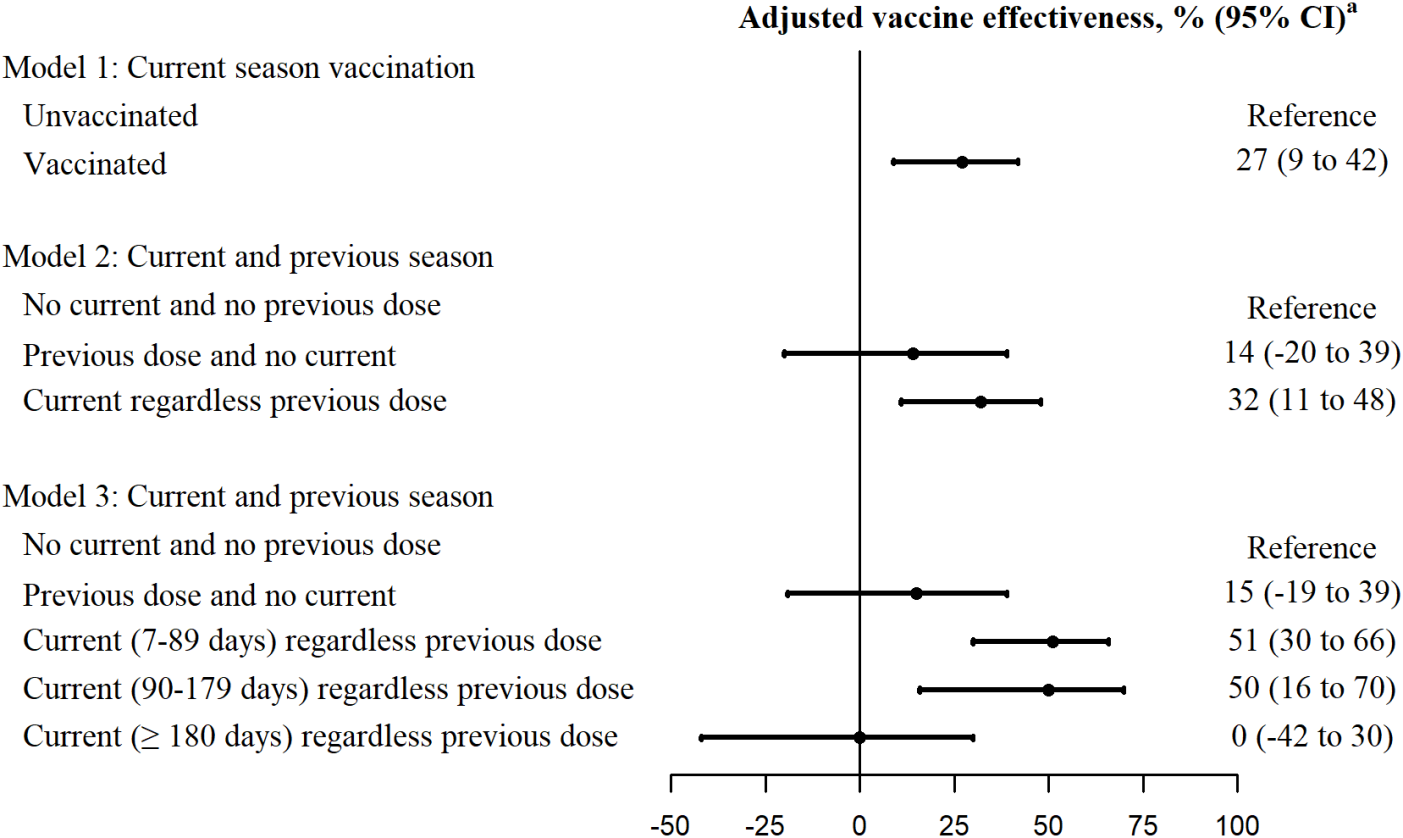
COVID‐19 vaccine effectiveness in preventing hospitalizations for confirmed COVID‐19 cases in Navarre, Spain, October 2023 to September 2024. Abbreviation: CI, confidence interval. ^a^Vaccine effectiveness adjusted by sex, age groups (45–64, 65–84, and ≥ 85 years), major chronic conditions, nursing home residence, and month of sample collection.

The overall adjusted estimate of the CVE in preventing hospitalizations due to COVID‐19 during the entire study period was 27% (95% CI, 9% to 42%) when only the current‐season vaccination was considered and increased up to 32% (95% CI, 11% to 48%) when patients with previous season vaccination were excluded from the reference category. The residual effect of previous season vaccination in preventing COVID‐19 hospitalizations among people unvaccinated in the current season was not statistically significant (14%, 95% CI, −20% to 39%). However, CVE estimates of current‐season vaccination were 51% (95% CI, 30% to 66%), 50% (95% CI, 16% to 70%), and 0% (95% CI, −42% to 30%) among people vaccinated 7 to 89, 90 to 179, and ≥ 180 days before COVID‐19 diagnosis, respectively (Figure 2 and Table 2).

**TABLE 2:** COVID‐19 vaccine effectiveness in preventing hospitalizations for confirmed COVID‐19 in Navarre, Spain, October 2023 to September 2024.

	Cases/controls	Crude vaccine effectiveness, % (95% CI)	Adjusted vaccine effectiveness,% (95% CI)^a^	*p*
**All hospitalized patients**				
*Model 1: Current season vaccination*				
Unvaccinated	194/1253	Reference	Reference	
Vaccinated	280/2324	22 (5 to 36)	27 (9 to 42)	0.005
*Model 2: Current and previous season*				
No current and no previous dose	108/723	Reference	Reference	
Previous dose and no current	86/530	−9 (−47 to 20)	14 (−20 to 39)	0.380
Current regardless previous dose	280/2324	19 (−2 to 36)	32 (11 to 48)	0.005
*Model 3: Current and previous season*				
No current and no previous dose	108/723	Reference	Reference	
Previous dose and no current	86/530	−9 (−47 to 20)	15 (−19 to 39)	0.344
Current (7–89 days) regardless previous dose	82/784	30 (5 to 48)	51 (30 to 66)	<0.001
Current (90–179 days) regardless previous dose	27/812	78 (66 to 86)	50 (16 to 70)	0.008
Current (≥180 days) regardless previous dose	171/728	−57 (−104 to −21)	0 (−42 to 30)	0.977
**Aged ≥ 65 years**				
*Model 1: Current season vaccination*				
Unvaccinated	162/938	Reference	Reference	
Vaccinated	264/2156	29 (12 to 44)	30 (12 to 45)	0.002
*Model 2: Current and previous season*				
No current and no previous dose	82/455	Reference	Reference	
Previous dose and no current	80/483	8 (−28 to 34)	21 (−13 to 44)	0.194
Current regardless previous dose	264/2156	32 (11 to 48)	38 (17 to 54)	0.001
*Model 3: Current and previous season*				
No current and no previous dose	82/455	Reference	Reference	
Previous dose and no current	80/483	8 (−28 to 34)	22 (−11 to 45)	0.163
Current (7–89 days) regardless previous dose	77/723	41 (18 to 58)	54 (32 to 69)	<0.001
Current (90–179 days) regardless previous dose	25/747	81 (70 to 88)	56 (26 to 74)	0.002
Current (≥180 days) regardless previous dose	162/686	−31 (−75 to 2)	11 (−30 to 39)	0.559

Abbreviation: CI, confidence interval.

^a^ Vaccine effectiveness adjusted by sex, age groups (45–64, 65–84, and ≥ 85 years), major chronic conditions, nursing home residence, and month of sample collection.

CVE estimate of the current‐season vaccination in adults aged ≥ 65 years was 38% (95% CI, 17% to 54%) on average of the year. The effect was higher (54%–56%) 7 to 179 days after vaccination and decreased to 11% after 180 days (Table 2).

Stratified analysis showed a moderate effect of COVID‐19 vaccination in the current season in preventing hospitalizations between October 2023 and March 2024 (50%, 95% CI, 28% to 65%); however, the effect almost disappeared from April to September 2024 (6%, 95% CI, −41% to 38%) (Table 3).

**TABLE 3:** COVID‐19 vaccine effectiveness in preventing hospitalizations for confirmed COVID‐19 by period in Navarre, Spain, October 2023 to September 2024.

	Cases/controls	Crude vaccine effectiveness, % (95% CI)	Adjusted vaccine effectiveness,% (95% CI)^a^	*p*
**October 2023 to March 2024**				
*Model 1: Current season vaccination*				
Unvaccinated	124/833	Reference	Reference	
Vaccinated	100/1378	51 (36 to 63)	48 (29 to 62)	<0.001
*Model 2: Current and previous season*				
No current and no previous dose	66/470	Reference	Reference	
Previous dose and no current	58/363	−14 (−66 to 22)	8 (−39 to 39)	0.682
Current regardless previous dose	100/1378	48 (28 to 63)	50 (28 to 65)	<0.001
*Model 3: Current and previous season*				
No current and no previous dose	66/470	Reference	Reference	
Previous dose and no current	58/363	−14 (−66 to 22)	8 (−39 to 49)	0.681
Current (7–89 days) regardless previous dose	82/778	25 (−6 to 47)	50 (26 to 66)	<0.001
Current (90–179 days) regardless previous dose	18/600	79 (63 to 87)	52 (9 to 74)	0.023
Current (≥180 days) regardless previous dose	—	—	—	
**April to September 2024**				
*Model 1: Current season vaccination*				
Unvaccinated	70/420	Reference	Reference	
Vaccinated	180/946	−14 (−54 to 15)	−4 (−44 to 25)	0.838
*Model 2: Current and previous season*				
No current and no previous dose	42/253	Reference	Reference	
Previous dose and no current	28/167	−1 (−69 to 40)	20 (−39 to 54)	0.431
Current regardless previous dose	180/946	−15 (−65 to 20)	6 (−41 to 38)	0.753
*Model 3: Current and previous season*				
No current and no previous dose	42/253	Reference	Reference	
Previous dose and no current	28/167	−1 (−69 to 40)	20 (−40 to 54)	0.438
Current (7–89 days) regardless previous dose	0/6	NA	NA	
Current (90–179 days) regardless previous dose	9/212	74 (46 to 88)	41 (−41 to 75)	0.233
Current (≥180 days) regardless previous dose	171/728	−42 (−104 to 2)	2 (−47 to 35)	0.910

Abbreviations: NA, not available; CI, confidence interval.

^a^ Vaccine effectiveness adjusted by sex, age groups (45–64, 65–84, and ≥ 85 years), major chronic conditions, nursing home residence, and month of sample collection.

In the sensitivity analysis maintaining the influenza‐positive patients in the control group, CVE estimates were ≤ 3 percentage points lower than the results of the main analysis (Table 4).

**TABLE 4:** Sensitivity analysis of COVID‐19 vaccine effectiveness in preventing hospitalizations, including influenza confirmed cases in the control group in Navarre, Spain, October 2023 to September 2024.

	Cases/controls	Crude vaccine effectiveness, % (95% CI)	Adjusted vaccine effectiveness,% (95% CI)^a^	*p*
**All hospitalized patients**				
*Model 3: Current and previous season*				
No current and no previous dose	108/823	Reference	Reference	
Previous dose and no current	86/593	−11 (−50 to 18)	13 (−20 to 37)	0.386
Current (7–89 days) regardless previous dose	82/987	37 (14 to 53)	49 (28 to 64)	<0.001
Current (90–179 days) regardless previous dose	27/848	76 (63 to 84)	47 (11 to 68)	0.015
Current (≥180 days) regardless previous dose	171/732	−78 (−131 to −37)	0 (−43 to 29)	0.991
**Aged ≥ 65 years**				
*Model 3: Current and previous season*				
No current and no previous dose	82/513	Reference	Reference	
Previous dose and no current	80/537	7 (−30 to 33)	20 (−13 to 44)	0.201
Current (7–89 days) regardless previous dose	77/917	47 (27 to 62)	53 (31 to 68)	<0.001
Current (90–179 days) regardless previous dose	25/783	80 (68 to 87)	54 (22 to 73)	0.004
Current (≥180 days) regardless previous dose	162/690	−47 (−96 to −10)	10 (−32 to 38)	0.592
**October 2023 to March 2024**				
*Model 3: Current and previous season*				
No current and no previous dose	66/568	Reference	Reference	
Previous dose and no current	58/425	−17 (−71 to 19)	6 (−40 to 38)	0.745
Current (7–89 days) regardless previous dose	82/981	28 (−1 to 49)	48 (23 to 64)	0.001
Current (90–179 days) regardless previous dose	18/635	76 (58 to 86)	47 (1 to 72)	0.045
Current (≥180 days) regardless previous dose	—	—	—	
**April to September 2024**				
*Model 3: Current and previous season*				
No current and no previous dose	42/255	Reference	Reference	
Previous dose and no current	28/168	−1 (−70 to 40)	20 (−39 to 54)	0.423
Current (7–89 days) regardless previous dose	0/6	NA	NA	
Current (90–179 days) regardless previous dose	9/213	74 (46 to 88)	41 (−40 to 75)	0.231
Current (≥180 days) regardless previous dose	171/732	−42 (−105 to 2)	3 (−47 to 36)	0.892

Abbreviations: NA, not available; CI, confidence interval.

^a^ Vaccine effectiveness adjusted by sex, age groups (45–64, 65–84, and ≥ 85 years), major chronic conditions, nursing home residence, and month of sample collection.

### 3.3 Impact of COVID‐19 Vaccination

From the previous results, it was estimated that the autumn 2023 booster dose of the COVID‐19 vaccine averted 111 hospitalizations in the target population of adults aged ≥ 45 years during the 2023–2024 season, which represents 50.9 admissions per 100,000 people or 19.0% of the hospitalizations that would be expected in the absence of this booster dose. While the seasonal booster dose averted 30.9% of the COVID‐19 hospitalizations in the winter epidemic wave, in the summer wave this percentage was only 4.4% (Table 5).

**TABLE 5:** Number and rate of COVID‐19 hospitalizations observed and prevented by COVID‐19 vaccination among target population of adults aged ≥ 45 years by period in Navarre, Spain, October 2023 to September 2024.

Hospitalizations due to COVID‐19	Winter wave	Summer wave	Entire seasonal period
	October 2023–March 2024	April–September 2024	October 2023–September 2024
**Expected without booster dose**			
Number of cases	324.0	261.5	585.5
Rate per 100,000 people	147.9	119.3	267.2
**Observed**			
Number of cases	224	250	474
Rate per 100,000 people	102.2	114.1	216.3
**Prevented by the booster dose**			
Number of cases	100.0	11.5	111.5
Rate per 100,000 people	45.7	5.2	50.9
Proportion of expected	30.9%	4.4%	19.0%

It was necessary to administer the 2023–2024 seasonal COVID‐19 vaccine to 963 persons of the study population to prevent one hospitalization due to COVID‐19.

## 4 Discussion

The effectiveness of autumn 2023 COVID‐19 vaccination in preventing COVID‐19 hospitalizations in Navarre was moderate in the winter wave of COVID‐19, decreased over time since vaccination, and tended to disappear in the summer wave. In summary, the overall effect of the 2023–2024 seasonal COVID‐19 vaccination was 32% in preventing COVID‐19 hospitalizations in the study period from October 2023 to September 2024. The effect in preventing COVID‐19 hospitalizations during the first 6 months was within the range of results from other studies in the same season (49%–62%) [8, 9, 10, 23, 24, 25].

The SARS‐CoV‐2 XBB.1.5 and other XBB lineages predominated during the first months of the 2023–2024 season and were early replaced by the BA.2.86 variant and then by the JN.1 and KP.3 variants [1]. In our study, most cases between January and July 2024 were sequenced as BA.2.86 subvariants. Although the vaccine contained the XBB.1.5 variant, the circulating viruses continued to accumulate spike mutations. Some studies suggested that the CVE estimates against these new mutations slightly declined compared with that observed against the original variants [26, 27, 28].

In contrast with the seasonal influenza epidemics that usually concentrate the cases in winter, COVID‐19 epidemics have been distributed all over the year, and epidemic waves in summer were not rare [1]. The decline of the vaccine effect over time suggests the difficulty of maintaining a good vaccine protection with a single annual booster over the epidemic waves of COVID‐19.

During the Omicron‐dominant phase of the pandemic, protection from vaccine‐induced immunity decreased a few months after vaccine boosting [11, 14]. In our study, the moderate effect of autumn COVID‐19 vaccination in the first 6 months was followed by null effectiveness after that. Two studies evaluated the 2023 COVID‐19 vaccination effectiveness in the winter epidemic wave; a European network reported a decline in CVE from 69% in the first 60 days to 40% after that [6], and a study in the United States found a decline from 42% to 19% after JN.1 lineage became dominant [11]. Other studies conducted in Canada and the United States evaluated the CVE against COVID‐19 hospitalizations among adults in the long term, and both reported that the vaccination did not protect ≥ 180 days after vaccination [23, 25]. These results could guide vaccination strategies with a spring booster to high‐risk groups in case of an expected increase in COVID‐19 circulation in summer.

In our study population, with 49% vaccination coverage, the autumn 2023 vaccine averted 19% of the hospitalizations due to COVID‐19 in the 2023–2024 season, which demonstrates that a higher impact of vaccination is possible by increasing vaccination coverage. This impact was higher in the winter wave (30.9%) and declined to only 4.4% in the summer wave. The estimated number of 963 vaccine doses necessary to prevent one hospitalization due to COVID‐19 in the target population provides useful information for vaccination programs and decision‐makers. This figure was significantly higher than the estimates for the 2021–2022 season, when SARS‐CoV‐2 circulation was much more intense [18, 29].

Our main analysis (Models 2 and 3) estimated the total benefit of the COVID‐19 vaccination for the people, considering the current and previous season vaccinations, while most studies evaluated only the effect of the current season vaccine, regardless of any preexisting effect due to vaccination in the previous season (Model 1) [6, 7, 8, 9, 10]. Our results show that this second approach slightly underestimates the benefit of COVID‐19 vaccination in the population, as has been reported in previous studies [30, 31]. When people unvaccinated in the current season who received a booster dose in the previous one were excluded from the reference category, the current CVE estimate increased slightly (32% vs. 27%). People vaccinated in the previous season but unvaccinated in the current season did not show a significant preventive effect, as had been reported in another study [10].

Patients who have been vaccinated against COVID‐19 have a higher likelihood of influenza vaccination, which could bias CVE estimates [21]. Since all patients included in our study were tested for COVID‐19 and influenza, we excluded influenza‐positive samples from the control group to reduce this possible bias. However, in the sensitivity analysis including influenza patients in the control group, slightly lower CVE estimates were observed, which are consistent with another study that adjusted for influenza vaccination to control this possible bias [10].

The strengths of the present study include the test‐negative case–control design according to a European protocol to estimate CVE in hospitalized patients [20]. All cases were laboratory‐confirmed for COVID‐19 and were compared with test‐negative controls recruited in the same setting before neither the patient nor the physician knew the laboratory result, a fact that improves comparability and reduces bias. Patients were recruited in hospitals from the same region and therefore with the same vaccination protocol, vaccine products, and circulating variants. To avoid vaccination information bias, the vaccination status was obtained from the online regional vaccination registry. This study evaluated the short‐ and long‐term effects, including the second epidemic wave during summer, providing an overview of the effect of the autumn 2023 COVID‐19 vaccination campaign during the entire 2023–2024 season. The underlying population‐based cohort allowed for estimating the impact of this vaccination.

Some limitations may affect this study. Our study was performed in a region where COVID‐19 vaccination was recommended for people aged ≥ 60 years and people from 6 months to 59 years old with major chronic conditions; only the XBB.1.5 monovalent vaccine was used during the autumn 2023 campaign, and the study population included adults aged ≥ 45 years with an indication of COVID‐19 vaccination; therefore, caution should be taken in making generalizations of our results to other countries with different target populations for COVID‐19 vaccination or where other types of vaccines were used. The statistical power was reduced in some analyses, as was the CVE in younger adults or the residual effectiveness of the previous dose. The SARS‐CoV‐2 XBB.1.5 variant predominated during the first months in the 2023–2024 season, and it was replaced by the BA.2.86 [2]; however, the data were not sufficient to estimate the CVE against each variant. Prior SARS‐CoV‐2 infection could affect CVE estimates [12, 32], but it has not been considered because of the difficulty in ascertaining all cases of this broadly extended infection. Since this study included patients with different chances for COVID‐19 vaccination due to their age or presence of comorbidities, all analyses were adjusted by sex, age, major chronic conditions, nursing home residence, and month of sample collection to control for these potential confounders [33]. CIs were not obtained for the estimates of the number of hospitalizations prevented by COVID‐19 vaccination and the number of people vaccinated necessary to prevent one hospitalization; therefore, these estimates should be used with caution.

## 5 Conclusion

During the 2023–2024 season in Spain, the effectiveness of the autumn 2023 COVID‐19 vaccination was moderate in preventing hospitalizations due to COVID‐19. The CVE estimates decreased after 6 months since vaccination and were null during the summer COVID‐19 wave. The doses administered in the previous season did not show a significant residual effect in preventing COVID‐19 hospitalizations. In a population with 49% vaccination coverage, the vaccine averted 19% of the COVID‐19 hospitalizations, demonstrating that a higher impact is possible by increasing the vaccination coverage and considering a spring booster if an important summer epidemic wave is expected. These results reinforce the recommendation for COVID‐19 vaccination in the target population to reduce the risk of severe COVID‐19.

## Author Contributions

**Iván Martínez‐Baz:** conceptualization, data curation, formal analysis, funding acquisition, investigation, methodology, project administration, software, supervision, validation, visualization, writing – original draft preparation, writing – review and editing. **Camino Trobajo‐Sanmartín:** data curation, investigation, project administration, writing – review and editing. **Miguel Fernández‐Huerta:** data curation, investigation, project administration, resources, writing – review and editing. **Ana Navascués:** data curation, investigation, project administration, resources, writing – review and editing. **Aitziber Echeverria:** data curation, investigation, project administration, writing – review and editing. **Nerea Egüés:** data curation, investigation, project administration, writing – review and editing. **Noelia Vera‐Punzano:** data curation, investigation, project administration, writing – review and editing. **María Eugenia Portillo:** data curation, investigation, project administration, resources, writing – review and editing. **Guillermo Ezpeleta:** data curation, investigation, project administration, writing – review and editing. **Jesús Castilla:** conceptualization, data curation, formal analysis, funding acquisition, investigation, methodology, project administration, software, supervision, validation, visualization, writing – original draft preparation, writing – review and editing.

## Ethics Statement

The Navarre's Ethical Committee for Research with Medicines approved the study protocol (PI_2024/150).

## Consent

Patient consent was waived due to the study analyzing epidemiological and microbiological surveillance data.

## Conflicts of Interest

The authors declare no conflicts of interest.

## Peer Review

The peer review history for this article is available at https://www.webofscience.com/api/gateway/wos/peer‐review/10.1111/irv.70163.

## Data Availability

The access to original data of this study requires authorization of the Navarre Health Department and approval by the Navarre's Ethical Committee for Research with Medicines.
